# Postbiotic effects elicited by heat-inactivated *Lacticaseibacillus rhamnosus GG* against cow’s milk allergy in human cells

**DOI:** 10.3389/fimmu.2025.1671729

**Published:** 2026-01-12

**Authors:** Franca Oglio, Lorella Paparo, Laura Carucci, Alessia Gaeta, Samantha Armiento, Serena Coppola, Antonio Molinaro, Cristina De Castro, Antonio Masino, Vittoria Mauriello, Marco Michelini, Marica Cozzolino, Rita Nocerino, Laura Pisapia, Roberto Berni Canani

**Affiliations:** 1 Department of Translational Medical Science, University of Naples Federico II, Naples, Italy; 2 ImmunoNutritionLab and NutriTechLab, University of Naples Federico II, Naples, Italy; 3 Department of Laboratory Medicine, Azienda Sanitaria Locale (ASL) Benevento, Benevento, Italy; 4 Institute of Genetics and Biophysics, Consiglio Nazionale delle Ricerche (CNR), Naples, Italy; 5 Department of Chemical Sciences, University of Naples Federico II, Naples, Italy; 6 Department of Biomedicine and Prevention, University of Rome “Tor Vergata”, Rome, Italy; 7 Task Force for Microbiome Studies, University of Naples Federico II, Naples, Italy; 8 European Laboratory for the Investigation of Food-Induced Diseases, University of Naples Federico II, Naples, Italy

**Keywords:** cytokines, food allergy, gut barrier, immune tolerance, LGG, Th2 response

## Abstract

**Background:**

The probiotic *L. rhamnosus* GG (LGG) elicits immunomodulatory actions facilitating the immune tolerance acquisition in children with cow’s milk allergy (CMA). Emerging data suggest that heat-inactivated LGG postbiotic (LGGp) could improve gut health and immune function. We investigated the tolerogenic actions elicited by LGGp against CMA in human cells.

**Methods:**

Peripheral blood mononuclear cells (PBMCs) collected from IgE-mediated CMA children (n=6, all Caucasian, mean age 31.2 months) were stimulated with beta-lactoglobulin (BLG) in the absence or presence of LGGp. Activated regulatory T cells (Tregs)number was assessed by flowcytometry. Growth factors and cytokines modulating interleukins (IL) production were assessed by RT-PCR. IL-4, 5, 13 and 10 production was assessed by ELISA. The LGGp effects on gut barrier were evaluated using a Caco-2 cells-based experimental model by measuring transepithelial electrical resistance (TEER); tight junction proteins, Mucin-2 (Muc-2), and lactase expression; and FITC dextran permeability,

**Results:**

LGGp exposure resulted in Tregs activation and beneficial modulation of cytokines production in PBMCs from CMA patients. These effects paralleled with beneficial effects on all biomarkers of gut barrier integrity.

**Conclusions:**

Our data suggest that LGGp, modulating several immune tolerance mechanisms, could be a promising therapeutic strategy against CMA.

## Introduction

Cow’s milk allergy (CMA) is one of the most common forms of food allergies, and of food-induced anaphylaxis in the pediatric age (1). It is also the most expensive allergic disease, and pediatric patients with CMA present an increased risk to develop other allergic manifestations later in the life (2–5). Cow’s milk allergy derives from alteration of immune tolerance mechanisms (6), mainly induced by alteration in gut barrier (7) enabling abnormal food allergens exposure to the immune system with subsequent Th2 cytokines release (8, 9). Evidence on the pivotal role elicited by gut microbiome in modulating immune tolerance mechanisms is supporting the use of probiotics for preventing or treating food allergy (10). *Lacticaseibacillus rhamnosus* GG (LGG) is one of the most studied probiotic strains for food allergy prevention and treatment (11–13). Preclinical and clinical data, from more than 2.200 pediatric patients with CMA evaluated in different countries, consistently demonstrated that LGG, alone or in combination with the extensively hydrolyzed casein formula, could promote a faster resolution of allergy-related gastrointestinal symptoms, could accelerate the acquisition of immune tolerance, and could reduce the occurrence of allergic march (5, 14). It has been demonstrated that LGG supplementation results in a beneficial modulation of gut microbiome composition and function with increased production of the tolerogenic short chain fatty acid butyrate in children with CMA (14, 15). The exact mechanisms of these beneficial actions are still not completely defined. Limitations to the use of probiotics are linked to stability, reduced shelf life and the potential risk of infection deriving from the use of live bacteria in vulnerable subjects (16). Indeed, the complete safety profile of probiotics for at-risk populations, including preterm neonates and immunocompromised individuals, is still debated. Their use may be associated with adverse effects, such as systemic infections and gastrointestinal symptoms (17). Postbiotics have recently attracted significant attention due to their promising potential to enhance host health. They are defined as inactivated probiotics, which means they are non-living microorganisms that no longer possess the ability to replicate or exert microbial activity. Additionally, postbiotics encompass any bioactive compounds produced during the metabolic processes of probiotics (16). These compounds include a variety of molecules, such as short-chain fatty acids, peptides, and cell wall components, which can offer health benefits to the host. Postbiotics can confer these effects in both direct ways, such as by influencing gut health or immune response, and indirect ways, such as by modulating the gut microbiota composition or enhancing nutrient absorption (16). Thanks to the absence of living microorganisms the limitations and risks associated with their use in pediatric nutrition are minimized compared to probiotics. Thus, it could be much easier, cheaper and safer adding postbiotics to pediatric dietary products, including infant formulas (18, 19).

The postbiotic action of LGG has been investigated for celiac disease and intestinal infections (20, 21). However, the postbiotic action of LGG in modulating immune tolerance in CMA is still largely unexplored. In this study we investigated whether LGG postbiotic (LGGp) could positively influence the immune tolerance mechanisms in human cells. These modulatory effects may lead to beneficial clinical outcomes for children with CMA. To achieve this, peripheral blood mononuclear cells (PBMC) collected from children with IgE-mediated CMA were stimulated with the major cow milk antigenic protein, beta-lactoglobulin (BLG), both in the absence or in the presence of LGGp. We assessed the expression of growth factors and cytokines modulating interleukins production, the rate of activated regulatory T cells (Treg), and the production of Th1 and Th2 cytokines. Additionally, human enterocyte (Caco-2) monolayers were used as a model of gut barrier. The LGGp effects on gut barrier integrity were evaluated by measuring transepithelial electrical resistance (TEER); tight junction proteins, Mucin-2 (Muc-2) and lactase expression; and by FITC dextran permeability.

## Materials and methods

### Preparation of the LGG postbiotic

*Lacticaseibacillus rhamnosus* GG (5x10^9^ CFU), obtained from ATCC (53103), was grown anaerobically, under sterile conditions to avoid any contamination, in 4 L of MRS medium (VWR chemicals) at 37°C, with shaking at 100 rpm overnight, and recovered by centrifugation (7.000 rpm for 15 min at 4°C). For the heat inactivation of LGG, the pellet (5.43 g) was suspended in 110 ml of water and heat-inactivated in autoclave at 80°C for 20 min. The full inactivation was verified by resuspending the pellet in MRS and adding it into different plates, at several decreasing concentrations (starting from 10–^1^ to 10^-7^). After 48 h at 37°C, no bacterial growth was observed in any of the plates used. After thermal inactivation, the solution was centrifuged (8.000 rpm x 10 min, 4°C) and the pellet, containing the heat inactivated LGGp, was washed twice.

The LGGp pellet was initially weighed and then resuspended in sterile Phosphate-Buffered Saline (PBS). This PBS stock was subsequently diluted to the final working concentration of 10 µg/ml using the specific cell culture media (RPMI for PBMCs and DMEM for Caco-2) for each experiment. This dose was determined in preliminary MTT assays (cytotoxicity tests) and dose-response experiments. The 10 µg/ml LGG postbiotic biomass dose corresponded to an initial concentration of 1x10^8^ CFU/ml of the original live LGG culture.

### Human peripheral mononuclear blood cells

Peripheral blood samples were obtained from IgE-mediated CMA pediatric patients (n=6, all Caucasian, mean age 31.2 months). Main demographic, anamnestic and clinical features of these patients are reported in **Table 1**. Blood samples were collected, stored, and analyzed in an anonymized manner with the permission of the Ethics Committee of the University Federico II of Naples (CE 315/20, 24/11/2020). Written informed consent was obtained from parents/tutors of each patient.

**Table 1:** Main demographic, anamnestic and clinical features of CMA patients evaluated into the study.

Features	CMA patients, n=6
Male, %	5 (83.3%)
Age, months (mean, SD)	31.2 (13.7)
Cesarean delivery, %	2 (33.3%)
Born at term, %	6 (100%)
Weight at birth, kg (mean, SD)	3.3 (0.4)
Breastfed for at least 2 months, %	6 (100%)
Weaning (months)	5 (1)
Siblings	1 (0.75)
Familial risk of allergy, %	4 (66.7%)
Age at CMA diagnosis, months (mean, SD)	6.3 (2.4)
Age, months (mean, SD)	31.2 (13.7)
Weight at CMA diagnosis, kg (mean, SD)	12.5 (3.1)
Length at CMA diagnosis, cm (mean, SD)	89.8 (11.8)
Positive specific serum IgE and/or prick by prick test for fresh milk, %	6 (100%)
Positive specific serum IgE and/or skin prick test for α-lactalbumin, %	5 (83.3%)
Positive specific serum IgE and/or skin test positive for β-lactoglobulin, %	6 (100%)
Positive specific serum IgE and/or skin prick test positive for casein, %	1 (16.7%)
Gastrointestinal symptoms at CMA onset, %	3 (50%)
Cutaneous symptoms at CMA onset, %	5 (83.3%)
Respiratory symptoms at CMA onset, %	1 (16.7%)

Continuous variables are reported as 50^th^ (median), and interquartile range (IQR) when not specified. Discrete variables are reported as the number and proportion of subjects with the characteristic of interest. CMA: cow milk allergy.

The PBMCs were isolated from 8 ml of heparinized peripheral blood by Ficoll density gradient centrifugation (Ficoll Histopaque-1077, Sigma, St. Louis, Missouri, USA). Briefly the blood was diluted 1:2 with PBS and layered on Ficoll gradient, centrifuged at 2000 rpm for 30 minutes at 18-20°C. After centrifugation, the opaque interface between plasma and Ficoll containing mononuclear cells was carefully aspirated with a Pasteur pipette and washed twice with 10 ml of PBS and centrifuged 10 min at 1400 rpm at room temperature. After, the PBMCs recovered were counted and plated at 2×10^5^ cells/well in 96-well plates in triplicate, in a final volume of 200 µl culture medium (RPMI 1640, Gibco) containing 10% FBS (Gibco), 1% non-essential amino acids (Gibco), 1% sodium pyruvate (Gibco), 1% penicillin/streptomycin (Gibco) and 1% of L-glutamine (Gibco).

### Human enterocyte cell lines

For all experiments, we used a well validated model of gut barrier based on Caco-2 cells monolayer (American Type Culture Collection, Middlesex, UK; accession number: HTB-37) (3, 22). Briefly, cells were grown in Dulbecco’s modified Eagle’s medium (DMEM; Gibco, Berlin, Germany) with a high glucose concentration (4.5 g/L) and L-glutamine, supplemented with 10% fetal bovine serum (FBS, Gibco) 1% non-essential amino acids (Gibco), 1% sodium pyruvate (Gibco), 1% penicillin/streptomycin (Gibco). The cells were incubated at 37°C in a humidified atmosphere containing 5% CO_2_. The culture medium was changed every 2 days.

### MTT cytotoxicity test

The MTT (3-(4,5-dimethylthiazol-2-yl)-2,5-diphenyltetrazolium bromide) test was conducted to evaluate the potential cytotoxic effects of LGGp at various concentrations. For these experiments, Caco-2 cells and PBMCs were seeded in 96-well cell culture plates and treated with different concentrations of LGGp (0.1, 1, 10, 100, and 1000 µg/ml) for 48 hours and for 4 days, respectively, at 37°C. Following the LGGp stimulation period, the cells were incubated with 10 μl of MTT solution (5 mg/ml in DMEM) for 2 hours. After incubation, the medium was removed, and the formazan crystals formed in the viable cells were dissolved in 100 μl of DMSO per well. The absorbance was measured at 490 nm using a microplate reader. Cells treated with only the medium served as the control.

### PBMCs stimulation protocol

The PBMCs from CMA pediatric patients were stimulated with beta-lactoglobulin (BLG; 200 µg/mL) in the presence or in absence of 10 µg/ml LGGp for 4 days. Best concentrations and timing were identified in preliminary dose-response and time-course experiments. Cells exposed to only medium were used as control. Afterward, the PBMCs were harvested for flow cytometry analysis and RNA was extracted to analyze the expression of growth factors and cytokines modulating interleukin (IL) production. Furthermore, culture supernatants were collected to assess IL-4, IL-5, IL-13, and IL-10 production.

### Tregs population analysis by flow cytometry

The Tregs were identified as CD4+/CD25+/FoxP3+ positive cells by flow cytometry analysis. The staining was performed using Foxp3/Transcription Factor Staining Buffer Set (eBioscience™ Cat.no 00-5523-00) and specific monoclonal antibodies: anti human Foxp3, anti-human CD25, anti-human CD4 (Cytosens). A total of 50,000 events were acquired for analysis, after gating lymphocytes based on the FSC/SSC dot plot. All phenotypes were analyzed with FACS Canto II system and data elaborated using the DIVA software (BD Biosciences, Milan, Italy).

### Assessment of cytokines production by PBMCs

The concentrations of IL-4, IL-5, IL-13, and IL-10 in PBMCs supernatant were measured using specific human ELISA assay kits (Elabscience Biotechnology Inc. Wuhan, Hubei). The minimum detection concentrations were 31.25 pg/ml for IL-4, 15.6 pg/ml for IL-13 and for IL-5, and 1.6 pg/ml for IL-10. The ELISAs were conducted according to the manufacturer’s recommendations.

### Human enterocytes stimulation protocol

Caco-2 cells were seeded 300.000 cells/well in a six well plate and stimulated after 15 days post-confluence with 10 µg/ml LGGp for 48 h. The best concentration and timing were identified in preliminary dose-response and time-course experiments. Cells exposed to only medium were used as control. Afterward, the supernatants were harvested and stored at −20°C for further use. The experiments were repeated 3 times in triplicate.

### Transepithelial electrical resistance

To evaluate the enterocytes monolayer integrity by TEER, Caco-2 cells (2x10^6^/well) were seeded on polycarbonate 6-well Transwell^®^ membranes (Corning, Life Science, Kennebunk, USA). After 15 days post-confluence, the TEER of the enterocyte’s monolayer was measured every 24 hours for a total of 72 hours, using an epithelial Volt-Ohm Meter (Millicel-ERS-2, Millipore, Billerica, MA, USA). The measured resistance value was multiplied by the area of the filter to obtain an absolute value of TEER, expressed as Ω cm^2^ and the TEER values were measured as follows: TEER = (measured resistance value−blank value) × single cell layer surface area (cm^2^).

### Quantitative real-time PCR

Total RNA was extracted from stimulated PBMCs and human enterocytes with TRIzol reagent (Gibco BRL, Paisley, UK). RNA samples were quantified using the NanoDrop 2000c spectrophotometer (Thermo Scientific) and purity was verified by A260/280 and A260/230 absorbance ratios. RNA reverse transcribed in cDNA with a High-Capacity RNA-to-cDNA™ Kit (Life Technologies, Waltham, MA, USA) according to the manufacturer’s instructions. Complementary DNA (cDNA) was stored at −80°C until use. In PBMCs stimulated with LGGp, the analysis focused on the expression of key genes associated with the upstream immune tolerance mechanism mediated by Tgfb1 (Hs00998133_m1), Ifna2 (Hs00265051_s1), Ptgs2 (Hs00153133_m1) and Csf2 (Hs00929873_m1). Quantitative real-time PCR (qRT-PCR) analysis was performed using Taqman Gene Expression Master Mix (Applied Biosystems, Vilnius, Lithuania) to evaluate the gene expression. In Caco-2 cells, the analysis evaluated genes crucial for barrier integrity and enterocyte differentiation, specifically Occludin (Hs05465837_g1) and ZO-1(Hs01551871_m1), Muc2 and Lactase. qRT-PCR analysis for Occludin and ZO-1 was performed using Taqman Gene Expression Master Mix (Applied Biosystems, Vilnius, Lithuania). While, gene expression of the mucin 2 (Muc2) and lactase were evaluated using a SYBR green Master Mix (Applied Biosystems, Grand Island, NY, USA). The primers used for Muc2 were forward (5’- CTCCGCATGAGTGTGAGT- 3’) and reverse (5’- TAGCAGCCACACTTGTCTG - 3’). The primers used for lactase were forward (5’-ACACGGTCGATTTCCTCTCT - 3’) and reverse (5’-TGGGTTCTTCATGGTGGAGG - 3’). The amplification protocol was 40 cycles of 15 s of denaturation at 95°C, 60 s of annealing at 60°C, and 60 s of elongation at 60°C in a Light Cycler 7900HT (Applied Biosystems, Grand Island, NY, USA). Data were analyzed using the comparative threshold cycle method. We used the glucoronidase beta (GUS-B) gene to normalize the level of mRNA expression (TaqMan probes: Hs00939627_m1; SYBR green Forward primer: 5’- GAAAATATGTGGTTGGAGAGCTCATT-3’; SYBR green Reverse primer: 5’-CCGAGTGAAGATCCCCTTTTTA - 3’).

### FITC-dextran permeability

To further investigate the effect of LGGp on gut permeability, the FITC dextran method was employed, as previously described (3). In brief, Caco-2 cells were seeded on a transwell plate and cultured for 15 days post-confluence to allow for differentiation. A complete DMEM solution containing 1 mg/mL of FITC dextran (Sigma-Aldrich, St. Louis, MO, USA) was added to the upper chamber of the transwell plate, while 1.5 mL of complete medium was placed in the lower chamber. The plate was incubated at 37°C for 24 hours. The fluorescence intensity of the medium in the lower chamber was measured at 15, 30, and 120 min and after 24 h using a fluorometer (Tecan, Infinite F200, Tecan Group Ltd. Männedorf, Switzerland) to determine the concentration of FITC dextran.

### Statistical analysis

A study monitor reviewed the data forms for completeness, clarity, consistency, and accuracy. All data were entered into the study database using a single data entry method by the same researcher. The study database was cleaned according to standard procedures and was locked before statistical analysis by the statistical team. The Kolmogorov-Smirnov test was used to determine whether continuous variables were normally distributed, in which case they were reported as mean and standard deviation (SD). Continuous variables that were not normally distributed were reported as median and interquartile range (IQR) with minimum and maximum. Categorical variables were reported as the number and proportion of subjects with the characteristic of interest. To evaluate the differences between continuous variables, the independent sample t test or Mann-Whitney U test were performed. The level of significance for all statistical tests was two‐sided, p<0.05. All analyses were performed using SPSS for Windows (SPSS Inc, version 23.0, Chicago, IL) and GraphPad Prism 10.

## Results

### Defining the best LGGp dose

Dose-response and time-course experiments revealed that the best effective dose for LGGp in both human PBMCs and enterocytes was 10 μg/ml. MTT experiments demonstrated that this LGGp dose was well tolerated by human PBMCs and enterocytes (**Supplementary Figure 1A, B**).

### Effects on tolerogenic mechanisms in human PBMCs

**Figure 1:**
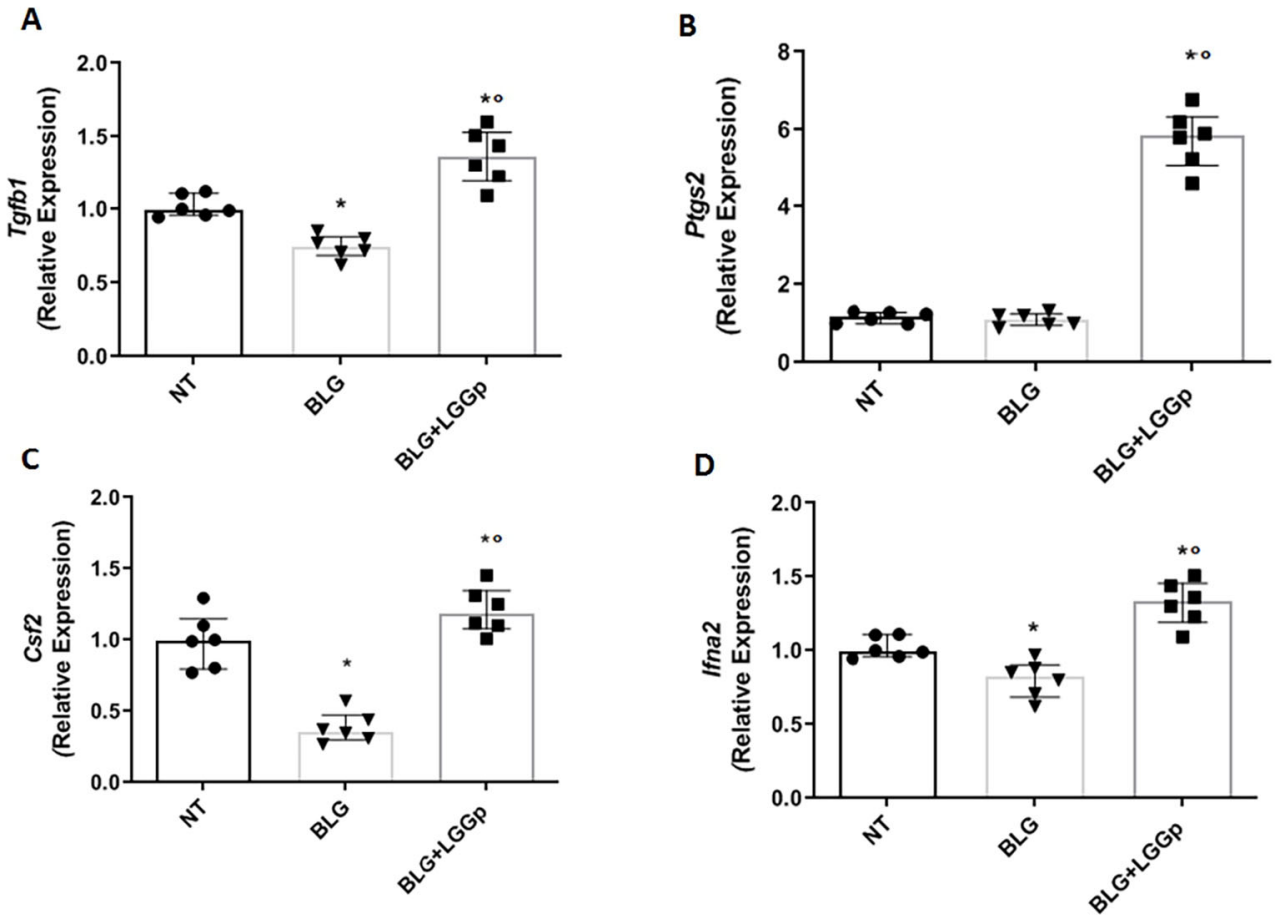
Effects of LGGp on the activation of regulatory growth factors and cytokines modulating interleukins production. Peripheral mononuclear blood cells (PBMCs) collected from six children affected by IgE-mediated CMA were exposed to 10 µg/ml LGGp for 4 days. Cells were harvested for RT-PCR assay. The LGGp significantly enhanced the expression levels of *Tgfb1***(A)**, *Ifna2***(B)**, *Ptgs2***(C)** and *Csf2***(D)**. Data are expressed as median and interquartile range of 6 independent experiments. Data were analyzed using Mann Whitney U test. LGGp= heat-inactivated LGG postbiotic; BLG, beta-lactoglobulin; *Tgf*b*1* (transforming growth factor β1), *Ifna2* (Interferon α2)*, Ptgs2* (Prostaglandin-Endoperoxide Synthase 2), and *Csf2* (Colony Stimulating Factor 2). *p<0.05 vs BLG; °p<0.05 vs BLG.

To see whether LGGp could modulate immune tolerance mechanisms, we firstly conducted experiments aimed at examining the expression of regulatory growth factors and cytokines. We observed that LGGp was able to increase the expression of Tgfb1, Ptgs2, Csf2, and Ifna2, as shown in **Figures 1A–D**. Then we moved to the evaluation of regulatory T cells (Tregs) activation and of the production of cytokines by PBMCs from children with IgE-mediated CMA. To do this, PBMCs were exposed to the major antigenic peptide, beta-lactoglobulin (BLG), both in the absence or in the presence of LGGp.

**Figure 2:**
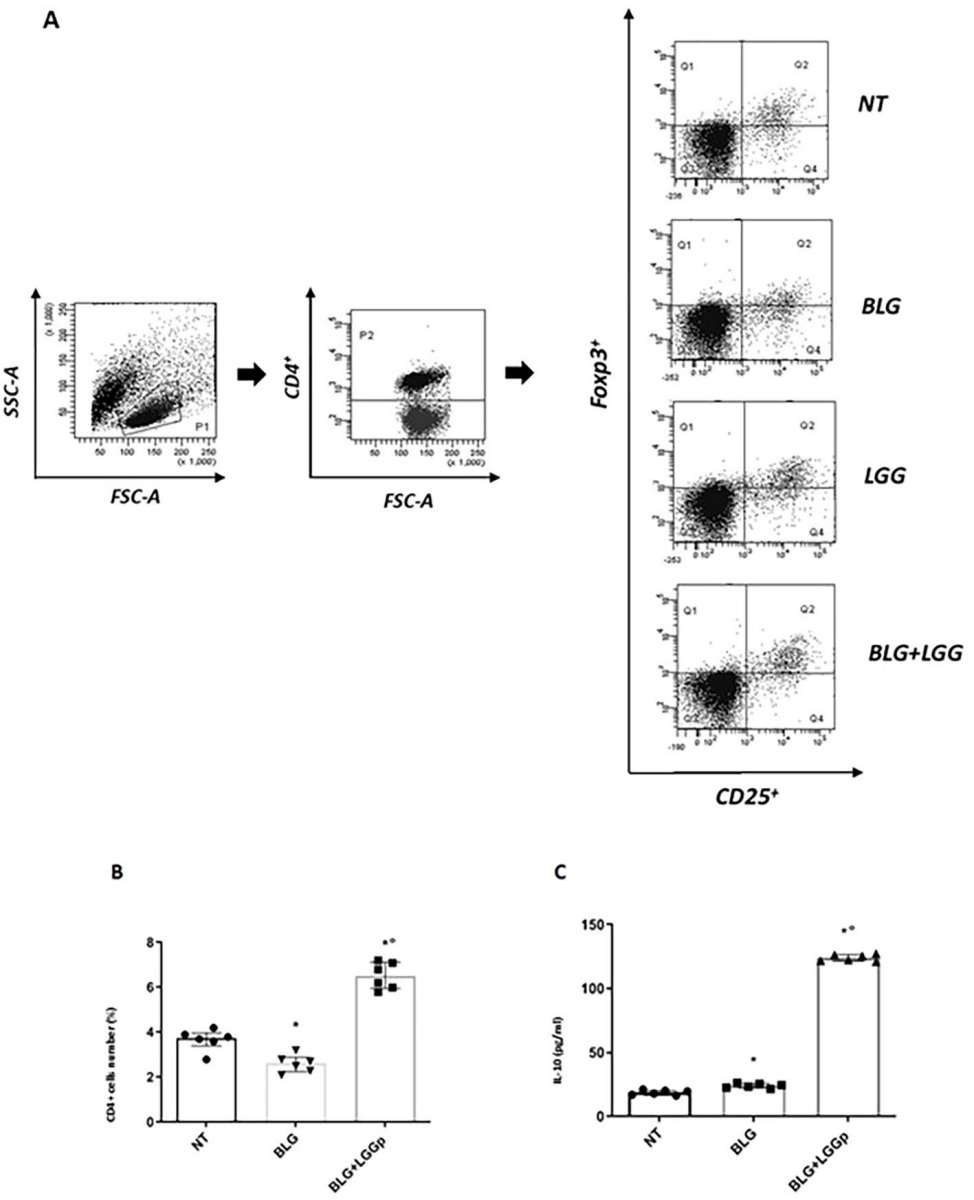
Effects of LGGp on tolerogenic mechanisms in human PBMCs from children affected by IgE-mediated CMA. Peripheral mononuclear blood cells (PBMCs) collected from 6 children with IgE-mediated CMA were exposed to 200 µg/ml BLG in the presence or in the absence of 10 µg/ml LGGp for 4 days. The BLG exposure was unable to significantly affect the number of CD4^+^/CD25^+^/Foxp3 **(A, B)** as well as the IL-10 production **(C)** in PBMCs from CMA children. The incubation with only LGGp or with LGGp+BLG resulted in an increase in CD4^+^/CD25^+^/Foxp3^+^ T cells number as well as in IL-10 production in PBMCs from CMA pediatric patients **(A–C)**. Representative dot plots obtained by flow-cytometry after staining with CD4+/CD25+/Foxp3 **(A)**. Data are expressed as median and interquartile range of 6 independent experiments. In Panel A, is reported the gating strategy applied for CD4^+^ Treg detection, using representative dot plots from one of the experiments summarized in the Results. First, lymphocytes were identified based on size and granularity using FSC-A and SSC-A parameters. Within this lymphocyte gate, CD4^+^ T cells were selected and subsequently analyzed in the final dot plot for CD25 and Foxp3 expression. The regulatory T-cell (Treg) population, defined as CD4+CD25^+^Foxp3^+^, is displayed in quadrant Q2 of the dot plots. Data were analyzed using Mann Whitney U test. LGGp, heat-inactivated LGG postbiotic; BLG, beta-lactoglobulin. *p<0.05 vs NT; °p<0.05 vs BLG.

We observed that LGGp significantly modulated the immune tolerance network by increasing the rate of activated Regulatory T cells (Tregs) in PBMCs from CMA children, as shown in **Figure 2A, B**. This Treg expansion, which represents a direct mechanism for tolerance induction, was accompanied by increased production of the tolerogenic and anti-inflammatory cytokine IL-10 (**Figure 2C**).

**Figure 3:**
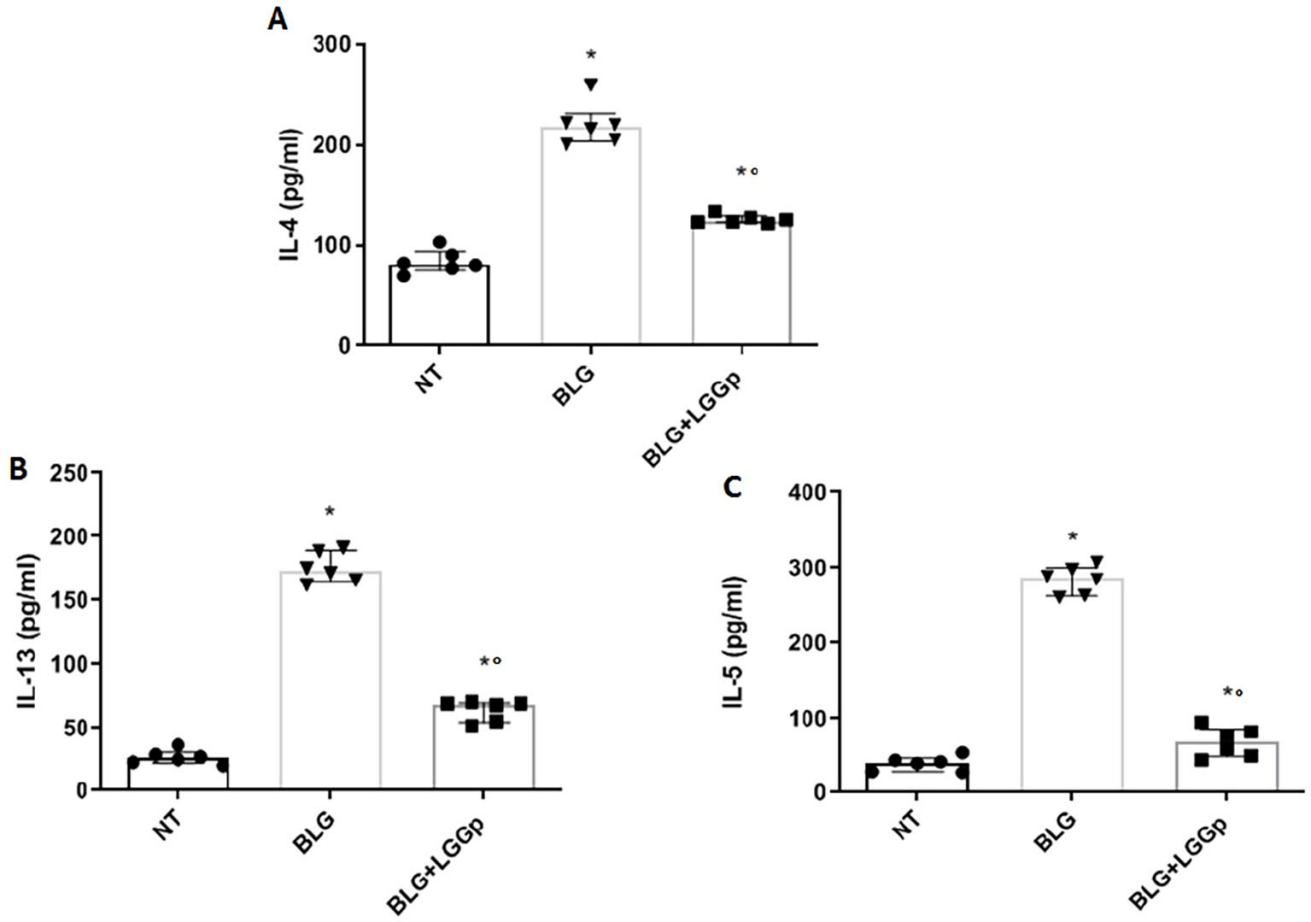
Effects of LGGp on Th2 cytokines response in human PBMCs from children affected by IgE-mediated CMA. Peripheral mononuclear blood cells (PBMCs) collected from six children affected by IgE-mediated CMA were exposed to 10 µg/ml LGGp for 4 days. The supernatants were collected for cytokines analysis. PBMCs stimulation with 200 µg/ml BLG resulted in a significant increase of Th2 cytokine production: IL-4 **(A)**, IL-13 **(B)** and IL-5 **(C)**. In the presence of LGGp, the BLG effects were blunted. Data are expressed as median and interquartile range of 6 independent experiments. Data were analyzed using Mann Whitney U test. LGGp, heat-inactivated LGG postbiotic; BLG, beta-lactoglobulin. *p<0.05 vs BLG; °p<0.05 vs BLG.

Lastly, we examined the production of Th2 cytokines by PBMCs exposed to BLG, both in the absence or in presence of LGGp. As expected, the incubation with BLG led to a significant increase in the production of Th2 cytokines by PBMCs, as shown in **Figures 3A–C**. Notably, the addition of LGGp significantly inhibited these effects, as shown in **Figures 3A–C**.

### Effects on gut barrier

**Figure 4:**
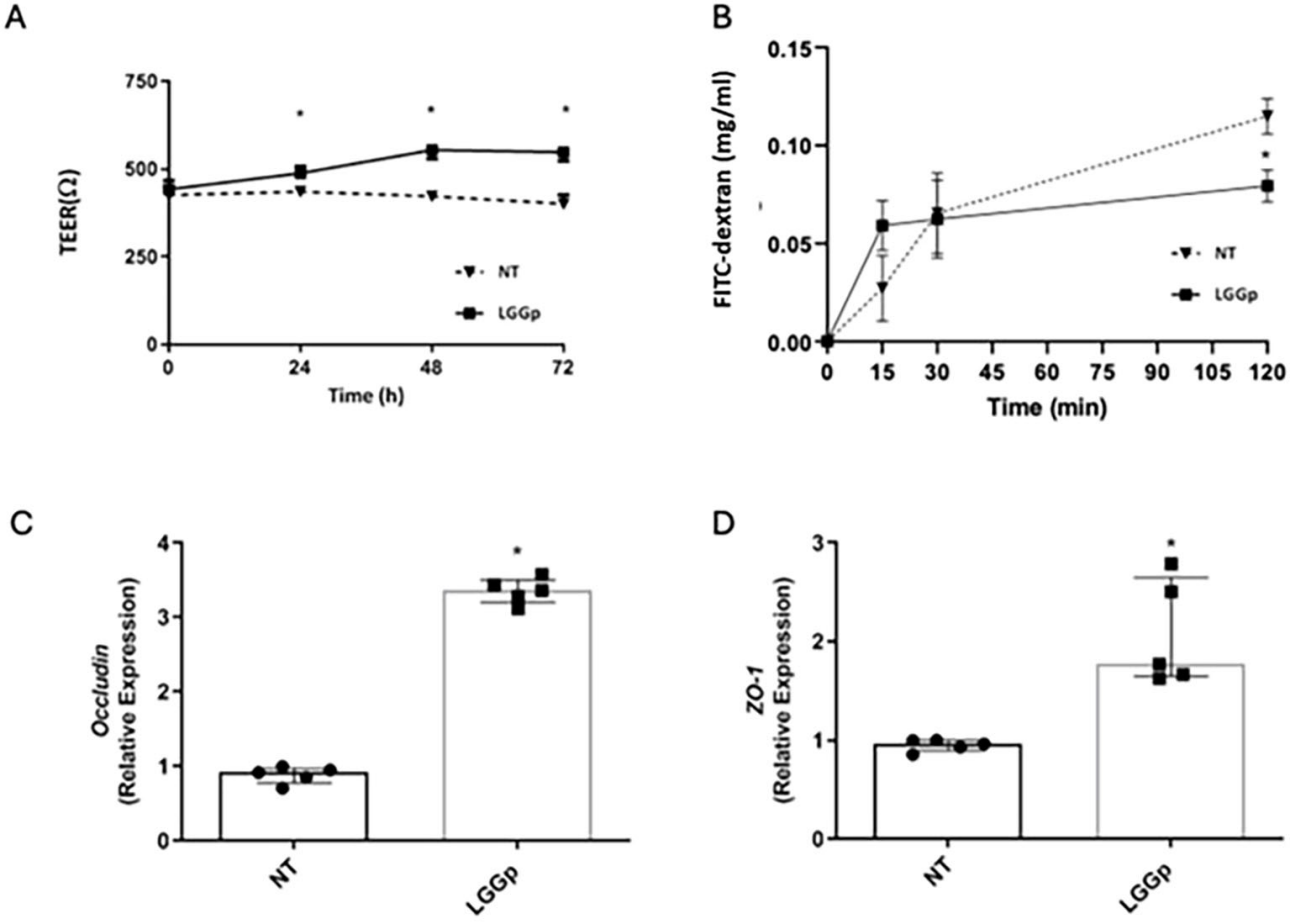
Effects of LGGp on gut barrier integrity. Caco-2 cells were stimulated with 10 µg/ml LGGp for 48 **(h)** The TEER values were measured as follows: TEER = (measured resistance value−blank value) × single cell layer surface area (cm^2^). The exposure to LGGp elicited a significant increase in TEER **(A)**. Caco-2 cells were stimulated with 10 µg/ml LGGp for 48 h (B, C, D). FITC dextran permeability was assessed in transwell plate **(B)** and appeared reduced after 2 h in cells pre-treated with LGGp. Cells were processed for mRNA analysis by RT-PCR. *Occludin***(C)** and *ZO-1***(D)** expression levels were significantly increased in Caco-2 cells exposed to LGGp. RT-PCR analysis was performed using the comparative threshold cycle (CT) method. Gene expression was normalized against the expression of the reference gene glucoronidase beta (GUS-B). Each point represents median and error with range **(A, B)** or median and interquartile range **(C, D)** of five independent experiments. Data were analyzed using Mann Whitney U test. LGGp, heat-inactivated LGG postbiotic; TEER, Trans-epithelial electrical resistance; ZO-1, zonula occludens 1. *p<0.05 *vs* NT.

**Figure 5:**
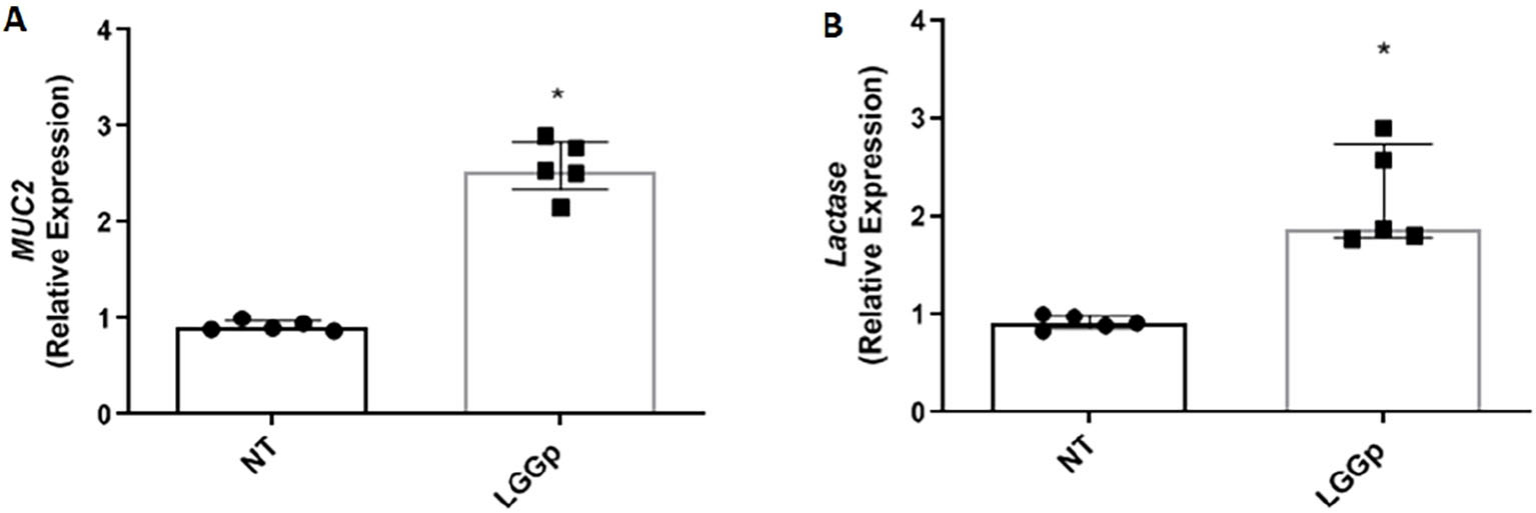
Effects of LGGp on human enterocytes differentiation. Caco-2 cells were stimulated with 10 µg/ml LGGp for 48 **(h)** Cells were processed for mRNA analysis by RT-PCR. *Muc2***(A)** and *Lactase***(B)** expression levels were significantly increased in Caco-2 cells exposed to LGGp. RT-PCR analysis was performed using the comparative threshold cycle (CT) method. Gene expression was normalized against the expression of the reference gene glucuronidase beta (GUS-B). Each point represents median and interquartile range of five independent experiments. Data were analyzed were analyzed using Mann Whitney U test. LGGp, heat-inactivated LGG postbiotic; Muc-2, mucin 2; *p<0.05 *vs* NT.

The incubation with LGGp resulted in a significant increase of TEER value as shown in **Figure 4A**. Additionally, we evaluated the expression of two major tight junction proteins, Occludin and ZO-1. Stimulation with LGGp resulted in a significant increase in the expression of both tight- junction proteins **Figures 4C, D**. To further assess the effects of LGGp on gut barrier integrity, we measured the gene expression levels of two biomarkers associated with mucus production (Muc2) and enterocyte differentiation (lactase). After 48 hours incubation with LGGp, we observed an increase in the expression levels of both Muc2 and lactase in human enterocytes (**Figures 5A, B**). Finally, these findings paralleled with a decreased gut barrier permeability induced by LGGp as demonstrated by the results of FITC-dextran experiments (**Figure 4B**).

## Discussion

We found that LGGp could regulate several mechanisms involved in the immune tolerance network. Previous evidence reported a tolerogenic action elicited by the probiotic LGG with a skewing toward Th1 response in a CMA mouse model (11), and in PBMCs from healthy donors (23). Another study demonstrated that the probiotic LGG could reduce IL-4 production, through a modulation of dendritic cells function, resulting in hypo-responsiveness of human Th2 cells (24).

Consistent with these findings, our results indicated that, also the LGGp could modulate immune tolerance, increasing the activation of Treg cells and IL-10 production and reducing the major Th2 cytokines (i.e., IL-4, IL-5, and IL-13) production in response to BLG exposure in PBMCs from children with CMA. The upstream mechanism of these LGGp effects could involve, at least in part, the activation of major regulatory molecules of immune function *Tgfb1, Ifna2, Ptgs2* and *Csf2* (25).

Furthermore, our findings suggest that LGGp could exert a positive effect on gut barrier integrity with an upregulation of TEER value, tight junction proteins, Muc2 and lactase expression, with a subsequent reduction in FITC dextran permeability.

These data resembled the results previously obtained by others using LGG supernatant or purified LGG soluble protein, named HM0539, showing a gut barrier protective function with increased mucus secretion and tight-junction proteins expression and reduced gut permeability in different animal models of infections, colitis and acute liver failure (26).

Clinical evidence in pediatric patients affected by CMA demonstrated that the living probiotic LGG, alone or in combination with the extensively hydrolyzed casein formula, could induce a faster resolution of the gastrointestinal symptoms and immune tolerance acquisition, with a preventive action against allergic march (5, 14). A beneficial modulation of gut microbiome composition and function, with increased abundance of butyrate-producers’ bacteria, was also observed in pediatric patients with CMA receiving a dietary treatment with extensively hydrolyzed casein formula supplemented with probiotic LGG (15).

Here we provide evidence on additional mechanisms elicited by LGG in facilitating immune tolerance though a direct interaction with human cells. Our data highlight a range of beneficial effects on immune tolerance, suggesting that LGGp could offer a safer therapeutic strategy for pediatric patients affected by CMA (16, 27).

Our data are well in line with other evidence obtained by others using different postbiotics products. For instance, Feng et al. demonstrated that heat-killed *L. plantarum* effectively alleviated allergy symptoms and regulated Th1/Th2 cell balance in rats with whey protein-induced food allergy (28). Miranda et al. demonstrated that administering heat inactivated *Akkermansia muciniphila* reduced levels of IgE antibodies against ovalbumin (OVA) and decreased eosinophil counts in a murine model of ovalbumin food allergy (29). Niu et al. showed how oral supplementation with a postbiotic from *Bifidobacterium longum*, could also mitigate allergic airway inflammation in a murine model, reducing IL-4, Il-5 and IL-13 levels, and modulating gut microbiome (30).

The strength of our study is mainly related to the use of relevant human primary cells (i.e., PBMCs from IgE-mediated CMA pediatric patients) and validated model of human intestinal epithelial cell monolayer (i.e., Caco-2) (3, 31, 32), increasing the translational relevance of our findings. Furthermore, we recognize as major limitations of our study the lack of co-culture model experiments using PBMC and Caco-2 to provide a more comprehensive and physiological assessment of the LGGp effects. Another relevant limitation could derive by the lack of investigation on LGGp-derived bioactive compounds. Previous data suggested the potential role of peptides produced by LGG in modulating the expression of tight junction proteins, and enterocytes differentiation (26, 33). Other studies demonstrated the ability of LGG-derived DNA sequence in modulating the expression of IL-4 in human cells (34, 35). Future studies are advocated to better define which component/s of LGGp could be involved in its beneficial protective action against CMA.

In conclusion, our results provided the first evidence on the beneficial modulatory action elicited by heat-inactivated LGGp on several mechanisms involved in immune tolerance in CMA. Our data suggest that this postbiotic has the potential to exert protective properties in the same way as the parent living LGG. These findings could open the way to the potential application of LGGp in clinical practice as a functional ingredient for the management of the most common form of food allergy in the pediatric age reducing limitations and costs of the actual LGG-supplemented dietary products.

## Data Availability

The raw data supporting the conclusions of this article will be made available by the authors, without undue reservation.
