# Supplementary material for: Postbiotic effects elicited by heat-inactivated Lacticaseibacillus rhamnosus GG against cow’s milk allergy in human cells
Source: Front Immunol. 2026 Jan 12;16:1671729. doi: 10.3389/fimmu.2025.1671729 (PMC12832425; doi:10.3389/fimmu.2025.1671729)

**Supplementary Figure 1.** **Cytotoxicity test**

Caco-2 cells and PBMCs were seeded in 96-well cell culture plates and treated with different concentrations of LGG postbiotics (0.1, 1, 10, 100, and 1000 µg/ml) for 48 hours and for 4 days, respectively, at 37°C. Following the treatment period, the cells were incubated with 10 μl of MTT solution (5 mg/ml in DMEM) for 2 hours. None of the doses administered resulted in a significant decrease in the viability of Caco-2 cells (panel A) and PBMCs (panel B).

Data are expressed as median and interquartile range of 6 independent experiments. Data were analyzed using Mann Whitney U test.

LGGp= heat-inactivated LGG postbiotic


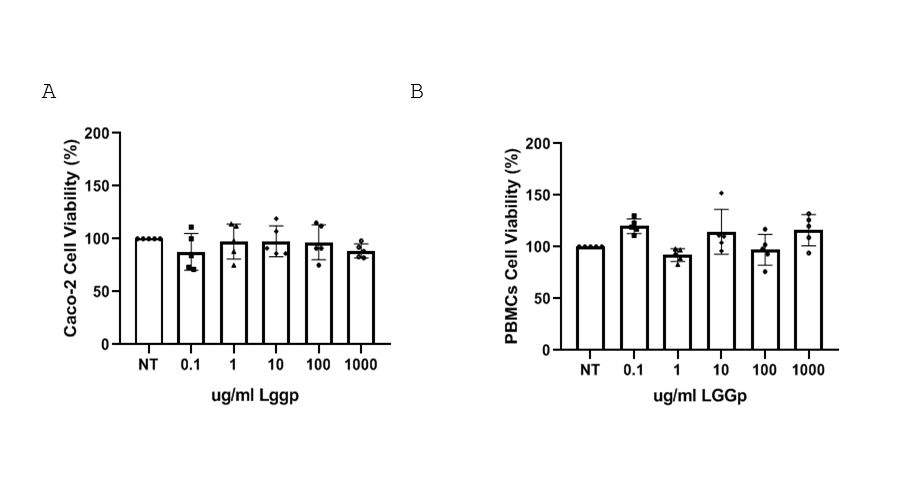

Supplement: Supplementary file 1 [file Table1.docx]
